# Prevalence of active transportation among adults in Latin America and the Caribbean: a systematic review of population-based studies

**DOI:** 10.26633/RPSP.2017.35

**Published:** 2017-03-23

**Authors:** Thiago Hérick de Sá, Leandro Fórnias Machado de Rezende, Maria Carolina Borges, Priscila Missaki Nakamura, Sebastian Anapolsky, Diana Parra, Fernando Adami, Carlos Augusto Monteiro

**Affiliations:** 1 Department of Nutrition, School of Public Health Universidade de São Paulo São Paulo Brazil Department of Nutrition, School of Public Health, Universidade de São Paulo, São Paulo, SP, Brazil.; 2 Department of Preventive Medicine, School of Medicine Universidade de São Paulo São Paulo Brazil Department of Preventive Medicine, School of Medicine, Universidade de São Paulo, São Paulo, SP, Brazil.; 3 Postgraduate program in Epidemiology Universidade Federal de Pelotas Pelotas, RS Brazil Postgraduate program in Epidemiology, Universidade Federal de Pelotas, Pelotas, RS, Brazil.; 4 Postgraduate program in Motor Science Universidade Estadual Paulista Júlio de Mesquita Filho Rio Clar SP Brazil Postgraduate program in Motor Science, Universidade Estadual Paulista Júlio de Mesquita Filho Rio Claro, SP, Brazil.; 5 Independent Consultant Independent Consultant Buenos Aires, BA Argentina Independent Consultant, Buenos Aires, BA, Argentina.; 6 Program in Physical Therapy, School of Medicine Program in Physical Therapy, School of Medicine St. Louis United States of America Program in Physical Therapy, School of Medicine, Washington University in St. Louis, St. Louis, United States of America.; 7 Laboratório de Epidemiologia e Análise de Dados Faculdade de Medicina do ABC Santo André, SP Brazil Laboratório de Epidemiologia e Análise de Dados, Faculdade de Medicina do ABC, Santo André, SP, Brazil.

**Keywords:** Urban health, healthy city, transportation, walking, motor vehicles, Argentina, Brazil, Colombia, Latin America, Caribbean Region, Salud urbana, ciudad saludable, transportes, caminata, vehículos a motor, Argentina, Brasil, Colombia, América Latina, Región del Caribe, Saúde da populaçâo urbana, cidade saudável, transportes, caminhada, veículos automotores, Argentina, Brasil, Colombia, América Latina, Regiâo do Caribe

## Abstract

***Objective*.:**

To describe the prevalence of “active” (self-propelled, human-powered) transportation in the Latin America and Caribbean (LAC) region over the past decade.

***Methods*.:**

MEDLINE, Excerpta Medica (Embase), SportDiscus, Lilacs, MediCarib, Web of Science, OVID, CINAHL, Scopus, Google Scholar, National Transportation Library, and TRIS/TRID were searched for articles on active transportation published between January 2003 and December 2014 with (at least) a title and abstract in English, Portuguese, or Spanish. Research was included in the study if the two reviewing authors agreed it 1) was conducted in an adult sample (≥ 18 years old), 2) was designed to be representative of any LAC area, and 3) reported at least one measure of active transportation. Reference lists of included papers and retrieved reviews were also checked. A total of 129 key informants (87 scientific experts and 42 government authorities) were contacted to identify additional candidate publications. Two other authors extracted the data independently.

***Results*.:**

A total of 10 459 unique records were found; the full texts of 143 were reviewed; and a total of 45 studies were included in the study, yielding estimates for 72 LAC settings, most of which were in Argentina, Brazil, and Colombia. No eligible studies were found for the years 2003–2004, resulting in a 10-year study time frame. Estimates were available for walking, cycling, or the combination of both, with a high degree of heterogeneity (heterogeneity index (I2) ≥ 99%). The median prevalence of active transportation (combining walking and cycling) was 12.0%, ranging from 5.1% (in Palmas, Brazil) to 58.9% (in Rio Claro, Brazil). Men cycled more than women in all regions for which information was available. The opposite was true for walking.

**Conclusions.:**

Prevalence of active transportation in LAC varied widely, with great heterogeneity and uneven distribution of studies across countries, indicating the need for efforts to build comprehensive surveillance systems with standardized, timely, and detailed estimates of active transportation in order to support policy planning and evaluation.

## RESUMEN

***Objetivo*.:**

Describir la prevalencia del transporte “activo” (autopropulsado o por tracción humana) en América Latina y el Caribe (ALC) durante el decenio pasado.

***Métodos*.:**

Se buscaron artículos sobre transporte activo publicados entre enero del 2003 y diciembre del 2014 que tuvieran (al menos) el título y el resumen en inglés, español o portugués en MEDLINE, Excerpta Medica (Embase), SportDiscus, Lilacs, MediCarib, Web of Science, OVID, CINAHL, Scopus, Google Scholar, National Transportation Library y TRIS/TRID. Los trabajos fueron incluidos en el estudio si los dos autores encargados de examinarlos coincidieron en que la investigación descrita 1) había utilizado una muestra de adultos (≥ 18 años de edad), 2) era representativa de una zona de América Latina y el Caribe y 3) consignaba al menos un parámetro relativo al transporte activo. También se verificó la bibliografía de los trabajos incluidos y las revisiones recuperadas. Se solicitó a 129 informantes clave (87 expertos científicos y 42 autoridades gubernamentales) que señalaran otras publicaciones que pudiesen reunir estos requisitos. Otros dos autores tuvieron a su cargo la extracción independiente de los datos.

Se encontraron 10 459 registros únicos; se examinaron los textos completos de 143 y se incluyeron en el estudio 45, que aportaron datos estadísticos sobre 72 entornos de América Latina y el Caribe, la mayoría de los cuales se encontraba en Argentina, Brasil y Colombia. No se encontraron estudios que reunieran los requisitos para los años 2003–2004, por lo que el periodo evaluado en el estudio fue de 10 años. Los trabajos incluidos aportaron datos estimativos sobre los traslados a pie, en bicicleta y la combinación de ambos tipos, con un alto grado de heterogeneidad (índice de heterogeneidad [I2] ≥ 99%). La mediana de prevalencia del transporte activo (traslados a pie y en bicicleta combinados) fue del 12,0%, dado el margen comprendido entre el 5,1% (en Palmas, Brasil) y 58,9% (en Río Claro, Brasil). Los hombres se transportan en bicicleta más que las mujeres en todas las zonas sobre las que se dispuso de datos, mientras que las mujeres se trasladan más a pie que los hombres.

**Conclusiones.:**

La prevalencia del transporte activo en América Latina y el Caribe es muy variable; se observa una gran heterogeneidad y una distribución desigual entre los estudios realizados en los distintos países, lo que indica la necesidad de emprender iniciativas para establecer sistemas integrales de vigilancia que aporten datos normalizados, oportunos y detallados acerca del transporte activo que sirvan de respaldo a la planificación y la evaluación de políticas. Salud urbana; ciudad saludable; transportes; caminata; vehículos a motor; Argentina; Brasil; Colombia; América Latina, Región del Caribe.

## RESUMO

Descrever a prevalência do “deslocamento ativo” (uso de modais de transporte autopropulsados e de propulsão humana) na região da América Latina e Caribe (ALC) na última década.

Foi realizada uma busca nos bancos de dados MEDLINE, Excerpta Medica (Embase), SportDiscus, Lilacs, MediCarib, Web of Science, OVID, CINAHL, Scopus, Google Scholar, National Transportation Library e TRIS/TRID por artigos sobre deslocamento ativo publicados entre janeiro de 2003 e dezembro de 2014 com (pelo menos) título e resumo em inglês, espanhol ou português. Pesquisas foram incluídas no estudo se os dois autores da revisão concordaram que a pesquisa 1) havia sido realizada em uma amostra de adultos (≥ 18 anos de idade), 2) tinha o intuito de ser representativa de uma área da ALC e 3) relatava pelo menos uma medida de deslocamento ativo. As referências bibliográficas dos artigos e revisões incluídos também foram analisadas. Foram contatados 129 informantes-chave (87 peritos científicos e 42 autoridades de governo) para identificar possíveis publicações adicionais de interesse. Outros dois autores extraíram os dados de maneira independente.

Foram encontrados 10 459 registros não duplicados; os textos completos de 143 foram examinados; e 45 foram incluídos na revisão, gerando estimativas para 72 regiões da ALC, a maioria na Argentina, Brasil e Colômbia. Não foi encontrado nenhum estudo dos anos 2003–2004 que atendesse os critérios de inclusâo; portanto, o período de análise foi de 10 anos. Foram obtidas estimativas para caminhada, deslocamento com bicicleta ou a combinação de ambos os modais; con alto grau de heterogeneidade (índice de heterogeneidade (I2) ≥ 99%). A prevalência mediana de deslocamento ativo (combinação de caminhada e deslocamento com bicicleta) foi de 12,0%, variando de 5,1% (em Palmas, Brasil) a 58,9% (em Rio Claro, Brasil). Homens andaram de bicicleta mais do que as mulheres em todas as regiões para as quais havia informações disponíveis. Constatou-se o oposto em relação à caminhada.

***Conclusões*.:**

A prevalência de deslocamento ativo variou muito na ALC, com grande heterogeneidade e distribuição desigual de estudos entre países. Isso indica necessidade de esforços para construir sistemas de vigilância integrais que proporcionem estimativas padronizadas, oportunas e detalhadas do deslocamento ativo para subsidiar a formulação e avaliação de políticas.

In September 2015, heads of state joined in the United Nations General Assembly to discuss the implementation of a new set of goals post-2015—the Sustainable Development Goals (SDGs)— designed to build upon the health gains obtained from the Millennium Development Goals (MDGs) experience (1). The new agenda includes thematic areas, such as city development and energy, and key components promoting holistic and integrated responses, designed to achieve a healthier future, including “safe, affordable, accessible and sustainable transport systems for all” (SDG 11, Target 11.2) (1). Sustainable transportation systems are also in line with several targets from health-specific SDG 3, such as halving the number of global deaths and injuries from road traffic, and substantially reducing deaths and illnesses from air pollution (1), highlighting the importance of cross-cutting cooperation.

“Active” (self-propelled, human-powered) transportation is a key component for the development of healthy sustainable environments as it provides health benefits as well as ancillary benefits related to greenhouse gas emissions (2, 3). Moreover, increasing active transportation levels is a key population-wide strategy to reverse the burden of noncommunicable diseases (NCDs), given the great potential of tackling physical inactivity levels through the transportation sector (4). This is particularly important for low- and middle-income countries (LMICs) because they have the highest burden of NCDs relative to other regions (5), reinforcing historical health inequities.

The Latin America and Caribbean (LAC) region, which has undergone rapid urbanization, includes many LMICs with significant challenges in terms of transportation and urban planning (6). Moreover, despite being, overall, the most urbanized region in the world, with 80% of the population living in cities (7), LAC includes countries at different levels of urbanization and at different stages in the mobility transition (6). In recent years, in several settings within the LAC region, there have been several attempts to improve certain features of the urban environment and to reduce the social and spatial segregation against the marginalized population (6, 8–10). These initiatives have great potential to promote or sustain walking and cycling.

However, monitoring of data on active transportation has been sparse in LMICs, particularly for walking and cycling, as these modes of transportation were traditionally relegated to a secondary role in both health and transportation research (11). Failing to promote and monitor different levels of walking and cycling as well as other forms of active transportation might jeopardize efforts supporting that agenda in the SDG era, as has already been learned from the MDGs experience, in which limitations arose related to a lack of data related to various criteria, including targets linked with sustainable healthy environments (e.g., Target C of MDG 7: “Ensure Environmental Sustainability”) (2).

Across and within countries the LAC region has 1) marked differences in active transportation and 2) varied capacity to promote changes in transportation systems and/or provide representative estimates of prevalence. Despite recent efforts to compile these estimates, particularly for cycling (12), most of the available information is limited to the proportion of trips taken by type of active transportation, which is not an optimum indicator for public health monitoring, with very few studies collecting data on the prevalence of active transportation across the region. To help fill this gap, the objective of this study was to describe the prevalence of active transportation in the LAC region over the past decade.

## MATERIALS AND METHODS

A systematic review of population-based studies reporting the prevalence of any type of active transportation in the LAC region was performed in accordance with PRISMA^8^ guidelines (13) and guidance for health care reviews developed by the University of York's Centre for Reviews and Dissemination (CRD) (York, England) (14). Active transportation was defined as any self-propelled, human-powered mode of transportation.

### Search strategy

MEDLINE (through PubMed), Excerpta Medica (Embase), SportDiscus, Lilacs, MediCarib, Web of Science, OVID (OVID journals, OVID books, CAB Abstracts, and EBM reviews), CINAHL, Scopus, Google Scholar, National Transportation Library, and TRIS/TRID were searched for articles on active transportation published between January 2003 and December 2014 with (at least) a title and abstract in English, Portuguese, or Spanish. The previous decade was selected as the study period to obtain up-to-date estimates that covered a time frame relevant for policy planning. Search terms included variations of the following terms in English, Portuguese, and Spanish: *epidemiology, prevalence, rate, active travel, active transportation, active commuting, urban mobility, running, walking, pedestrian, cycling, bicycle, bike, paddling, rowing, travel survey, transport survey, demand survey, origin and destination, mobility survey, time-use survey, adult, men, women, Latin America, Central America, South America, Caribe,* and names of LAC countries. Searches included truncated and full-text terms as well as MeSH (medical subject heading) descriptors and their equivalents. The search strategy was adapted from the search of Saunders et al. (15), then adjusted for each database used, and is available from the corresponding author (THS).

The reference list of all selected manuscripts was reviewed and relevant reviews identified through the search and selection processes. A total of 129 key informants (87 scientific experts and 42 government authorities) were contacted by email to find any additional studies (including unpublished or ongoing research) that might be relevant for the review. All contacts by email included an initial email, followed by, in the case of no response, two follow-up emails sent within two and three weeks respectively. When email was not available, the key informants had their website searched for the same purpose (to find additional studies). Duplicate records were removed using EndNote™ online (formerly EndNote Web) (Thomson Reuters, Carlsbad, California, United States).

### Study selection

Two of the authors (THS and PMN) reviewed the search output and independently identified potentially relevant studies by reading titles and abstracts. Full-text articles were obtained either through online databases or through experts and authorities and selected based on the reviewers' consensus according to the following inclusion criteria: 1) reported original data of any active transportation type or of a combination of types (data from the first evaluation of any longitudinal study was included); 2) conducted in Latin America or the Caribbean (i.e., studies with Latin American or Caribbean populations living in other regions were not included); 3) had a sample designed to be representative of a particular area; and 4) reported estimates from the adult general population (≥ 18 years old). Disagreements between the two reviewers about which studies to include based on these inclusion criteria were solved by a third author/reviewer (LFMR).

### Data extraction and quality appraisal

Two authors (LFMR and FA) independently extracted the data using a data collection form pretested on a sample of papers. Another author (MCB) solved any disagreements related to the data extraction. For each study, information on the following variables was entered into the form: study design; methodological aspects; outcomes (as prevalence, or proportion of trips; if these were not available or possible to calculate, other measures were extracted—e.g., mean time); population characteristics; and study setting. Studies for which more than one paper was obtained had their data extracted from all manuscripts retrieved.

The same two authors (LFMR and FA) also evaluated the quality of the studies, using a previously agreed-upon protocol for assessment of quality and risk of bias (**Supplementary Material Appendix**). The criteria on the standardized checklist included the following: study presented a definition of active transportation; active transportation prevalence was one of the study's main objectives; target population, sampling strategy, data collection, and statistical analysis were well-defined/described; total population and response rates were reported; and analysis included key estimates, such as confidence intervals (CIs) or standard errors.

### Data analyses

**FIGURE 1.**
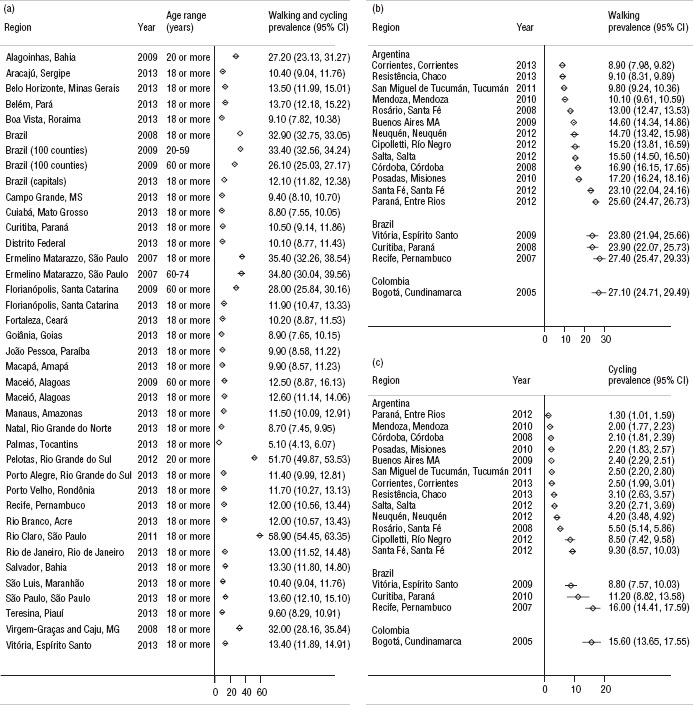
Prevalence of active transportation, including confidence intervals (CIs), for: a) walking and cycling combined, b) walking only, and c) cycling only, Latin America and the Caribbean, 2005–2014

***Source***: Prepared by the authors based on the results of the systematic review.

**FIGURE 2.**
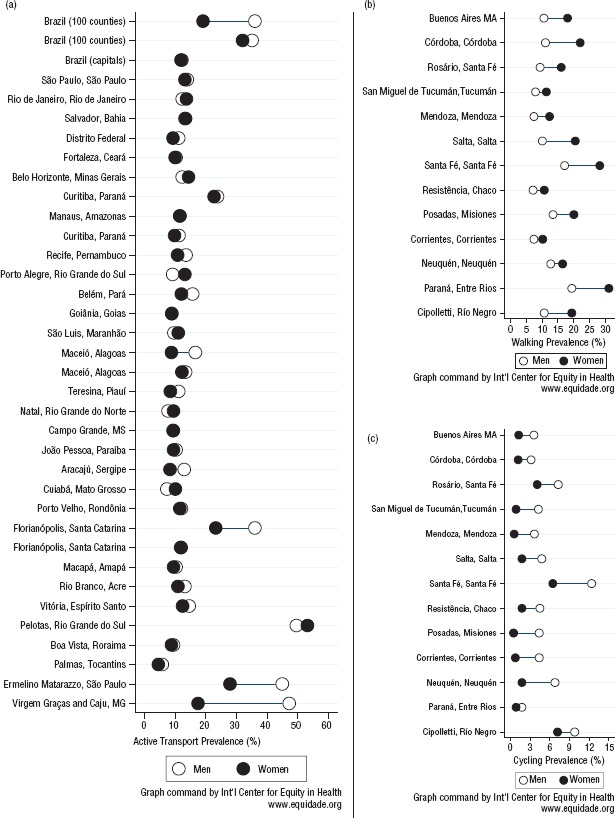
Difference in male–female prevalence for active transportation, including a) walking and cycling combined, b) walking only, and c) cycling only, Latin America and the Caribbean, 2005–2014

***Source***: Prepared by the authors based on the results of the systematic review.

Overall estimated prevalence of active transportation from each study (Figure 1) and estimated prevalence of active transportation stratified by commuting mode and sex (Figure 2) were depicted with forest plotting. In the case of multiple estimates for the same study population, only the most recent estimate was considered. When these estimates were not included in the original study, CIs were calculated using the standard error or data on prevalence and sample size. Median prevalence and range of active transportation by active transportation type (walking, cycling, or both combined) were also plotted (Figures 1b–1c and Figures 2b–2c). High heterogeneity across studies was defined as a heterogeneity index (I2) ≥ 50%, and a meta-regression model was selected to assess the sources of heterogeneity. Characteristics and quality of the studies were presented in narrative form and in relative frequencies. All analyses were performed in Stata 12.0 (StataCorp, College Station, Texas, United States).

## RESULTS

A total of 10 459 records were retrieved from the electronic database search (MEDLINE: 2 761, Embase: 507, Sport-Discus: 133, Lilacs and MediCarib: 2 946, Web of Science: 1 172, OVID: 1 239, CINAHL: 192, Scopus: 1 302, Google Scholar: 76, National Transportation Library: 83, TRIS/TRID: 48). Additional records were identified from reference lists (a total of 13), scientific experts (total of 35), and government authorities (8 published reports and 3 unpublished reports). All reports identified as coming from a government authority came from a single source. A total of 45 studies met the eligibility criteria (10, 16–54). The characteristics of the 45 eligible studies are summarized in Table 1. Studies that included more than one location or more than one period of analysis were initially presented separately, which resulted in 72 units of analysis, as shown in the **Supplementary Material Table**. Studies that estimated the prevalence of active transportation for population subgroups (e.g., adults versus elderly) and did not provide enough information for combining stratum-specific estimates were also presented separately. No eligible studies were found for the years 2003–2004, resulting in a 10-year study time frame.

Most studies were conducted in Brazil (28 or 62.2%), in urban areas (36 or 80.1%), and between 2005 and 2009 (27 or 60.0%). The majority of studies had a cross-sectional design (44 or 97.8%), sample size greater than 1 000 individuals (32 or 71.0%), used ≥ 150 min/week as the criterion for defining active transportation (15 or 33.3%), and used the International Physical Activity Questionnaire, long form (IPAQ-LF)^9^ (22 or 48.9%). The response rate was not reported in 23 (51.2%) of the studies (Table 1).

Estimates were only found for walking and cycling (i.e., none were available for any other form of active transportation, such as running or paddling). The prevalence of active transportation, combining walking and cycling modes, varied widely (I2 ≥ 99.9%) and was available only for Brazilian studies. Most Brazilian settings had at least one estimate from Brazil's telephone-based survey system, VIGITEL.^10^ The median prevalence of active transportation was 12.0%. The lowest prevalence was found in the capital city of Palmas, in the state of Tocantins in northern Brazil (5.1%; 95% CI: 3.4–6.8) and the highest was found in the city of Rio Claro, in the highlands of east-central Säo Paulo State in southeast Brazil (58.9%; CI: 54.5–63.3) (Figure 1a).

Few studies reported mode-specific prevalence of active transportation (17 for cycling and 17 for walking) and most of the data (13 studies) came from Argentine studies. Both cycling and walking prevalence were highly heterogeneous across studies (I2 ≥ 99%). The median prevalence of walking was 15.5%, ranging from 8.9% (CI: 8.0–9.8) in Corrientes, Argentina, to 27.1% (CI: 24.7–29.5) in Bogotá (Figure 1b). The median prevalence of cycling was 3.2%, ranging from 1.3% (CI: 1.0–1.6) in Paraná, Argentina, to 16.0% (CI: 14.4–17.6) in Recife, the capital city of the state of Pernambuco in northeast Brazil (Figure 1c).

Pooled prevalence for active transportation was not estimated due to the high levels of heterogeneity across studies. In addition, direct comparison of active transportation prevalence from different settings was challenged by the multiple sources of heterogeneity among studies, including the different cutoff points used to define active transportation (e.g., ≥ 10 min/week versus ≥ 150 min/week).

In relative terms, the gender gap varied broadly and in both directions for the estimates combining walking and cycling modes, with male–female prevalence ratios ranging from 0.7 (in the capital cities of Porto Alegre and Cuiabá in the Brazilian states of Rio Grande do Sul and Mato Grosso respectively) to 1.9 (in the capital city of Maceió in the coastal state of Alagoas) and 2.7 (in Virgem das Graças and Caju, villages in the Brazilian state of Minas Gerais) (Figure 2a). Mode-specific prevalence of active transportation by sex, which was only available for Argentine cities, consistently showed more walking among women and more cycling among men. All estimates for Argentine settings (n = 13) came from the transportation sector. Male–female prevalence ratios for walking ranged from 0.8 (in Neuquén, a province in western Argentina) to 0.5 (in Salta and Córdoba, cities in northwest and central Argentina respectively) (Figure 2b). Male–female prevalence ratios for cycling ranged from 1.4 (in Cipolletti, a city in the northern part of Argentine province Río Negro) to 8.8 (in Posadas, capital city of the Argentine province of Misiones) (Figure 2c). Age differences within studies were only available for walking and cycling separately and were systematically against the elderly population (**Supplementary Material Table**).

**FIGURE 3.**
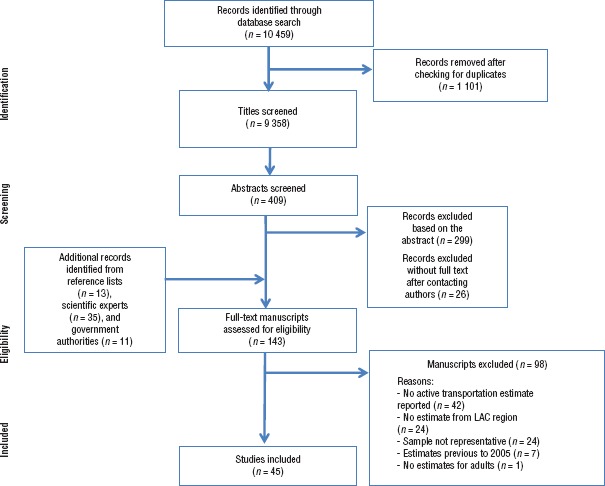
Flowchart of study selection for systematic review of research on active transportation, Latin America and the Caribbean, 2005–2014

***Source***: Prepared by the authors based on the systematic review process.

The results of the assessment of the studies' quality and risk of bias included the following: 1) no studies presented a definition of active transportation; 2) 87.2% included active transportation prevalence as one of their main objective; 3) 64.1% did not include CIs or standard errors for their prevalence estimates; 4) about 30% did not report or properly define the population, response rate, and/or sampling strategy; and 5) 20% did not include a well-described statistical analysis or the total population (**Supplementary Material Appendix**). Five studies were not assessed for quality or risk of bias because (although they met the eligibility criteria) the full-text reports were not published until July 2015. A detailed flowchart for the study review process is shown in Figure 3.

**TABLE 1.** Characteristics of active transportation studies, Latin America and the Caribbean, 2005–2014

Characteristic	No.	%
Country		
Argentina	13	29.0
Brazil	28	62.2
Chile	1	2.2
Colombia	2	4.4
Jamaica	1	2.2
Type of community		
Urban	36	80.1
Rural	1	2.2
Urban and rural	2	4.4
NR^a^	6	13.3
Year of data collection		
2005–2009	27	60.0
2010–2014	18	40.0
Study design		
Cross-sectional	44	97.8
Longitudinal	1	2.2
Sample size		
≤ 1 000	13	29.0
> 1 000	32	71.0
Active transportation criteria		
No/Yes	18	40.0
≥ 10 min/week	3	6.7
≥ 150 min/week	15	33.3
≥ 30 min/day	4	8.9
Other	5	11.1
Data collection instrument		
IPAQ^b^ (long form)	22	48.9
GPAQ^c^	2	4.4
VIGITEL^d^ questionnaire	6	13.3
Travel diaries	13	29.0
Other questionnaires	2	4.4
Response rate (%)		
60–80	11	24.4
80–100	11	24.4
NR	10	22.2
NA^e^	13	29.0
Total	45	100.0

***Source:*** Prepared by the authors based on the systematic review.

a NR: Not reported.

b IPAQ: International Physical Activity Questionnaire (developed by an International Consensus Group in 1998 as a surveillance instrument to measure multiple domains of physical activity).

c GPAQ: Global Physical Activity Questionnaire (developed by the World Health Organization (WHO) in 2002 as part of the WHO STEPwise Approach to Chronic Disease Risk Factor Surveillance for observation of physical activity).

d VIGITEL: Brazil’s Telephone-based Surveillance of Risk and Protective Factors for Chronic Diseases (Vigilância de fatores de risco e proteção para doenças crônicas por inquérito telefônico), which is based on the U.S. Behavioral Risk Factor Surveillance System (BRFSS).

e NA: Not applicable.

## DISCUSSION

This study conducted an extensive review of the literature to estimate the prevalence of active transportation in the LAC region. The findings show that estimates of active transportation and gender differences vary widely in the region for all forms of active transportation reported in the study sample (walking, cycling, and a combination of both). A lack of information about active transportation was observed for many LAC sites, with the available evidence concentrated in just a few countries. Due to 1) Argentina's integration of its public health and transportation sector agendas and 2) Brazil's provision of timely active transportation estimates through one of its health surveillance systems, those two countries were well represented in the study. Substantial methodological variation was found across studies, mainly in the data collection instrument and active transportation criteria.

Overall, the median prevalence of active transportation in the LAC region is low (15.5% for walking, 3.2% for cycling, and 12.0% for a combination of both variables) and below the prevalence in China and in most developed countries, even if only considering trips to work (55, 56). The LAC settings with the highest regional prevalence had rates much lower than the highest-prevalence settings in other regions (e.g., 16.0% for cycling in Recife, Brazil, versus 63.6% in the Netherlands (56)), except for the estimates combining walking and cycling. However, comparisons of information from other countries and settings are challenging because there is no standardization of instruments and indicators worldwide.

Gender differences in cycling and walking found in Argentinian cities might be explained by differences in trip characteristics, as women are more likely to make chauffeuring /accompanying, multipurpose, and/or encumbered trips, which are all less suited for cycling (and more suited for walking) than trips performed alone and unencumbered (57). The gender gap in cycling against women has been consistently observed in other places without a strong cycling culture (56, 58), and might be also related to infrastructural preferences and cultural norms, including greater risk aversion among women (57), out-group stereotypes, and experiences of marginalization (58). Higher car and motorcycle use by men, which is inversely correlated with active transportation in LAC settings (6, 49), also helps explain the gender gap in walking against men. As expected, age differences against the elderly were found for both walking and cycling, potentially reflecting the consequences of an environment less supportive for active transportation among vulnerable groups (36, 50).

### Limitations

This review had several limitations. First, despite the extensive search, which included 12 databases and research in the three most common languages in the region (English, Portuguese, and Spanish), active transportation estimates could not be found for many LAC populations. Second, there was a very low response from government authorities (1 out of 42 contacts). This single response, which provided estimates from 13 Argentine cities, highlighted the potential for multi-sectoral work related to the sustainable transportation systems agenda in that country, but also precluded the possibility of exploring variability in estimates through a multivariate meta-regression model, due to the scarcity of data and redundancy of the data source. Third, all included studies failed to present an active transportation construct, which contributed to the difficulty in assessing the prevalence of people in the LAC region engaging in active transportation. Finally, there was no assessment of either quality or risk of bias in any of the studies that were retrieved, underscoring the need for better study design and transparency of reporting.

### Recommendations

Author recommendations include standardizing measures, after the development of a construct for what constitutes active transportation—a challenge for public health and transportation researchers. In addition, the use of devices such as mobile GPS tracking products and accelerometers to objectively measure active transportation would be much welcomed. Periodical large cross-sectional surveys from more countries in the LAC region (in both rural and urban populations) would benefit 1) sustainable transportation planning in the SDG era and 2) natural experiment research that could help clarify how environmental changes influence the distribution of active transportation. Longitudinal studies examining determinants of active transportation in the LAC region are also needed. Qualitative analyses investigating cultural norms, infrastructure preferences, and travel patterns would provide insight on equity issues and facilitate improvements in multi-sectoral collaboration.

Although walking, cycling, and other forms of active transportation are not explicitly included as indicators in any goal in the SDG finalized text (1), self-propelled, human-powered transportation can be considered an indicator of integrated health and environmental sustainability (2), and cuts across a number of thematic areas, such as energy, city, health, and sustainability. Several transportation and urban planning interventions taking place in the region have the potential to favor active transportation (6, 8–10).

### Conclusions

Based on the results of this review, prevalence of active transportation in LAC varies widely, with great heterogeneity and uneven distribution of studies across countries. LAC local authorities should be encouraged to build comprehensive surveillance systems upon existing sources of information (e.g., health systems and transportation databases) to generate standardized, timely, detailed estimates of active transportation that can support policy planning and evaluation. This type of data could help leverage active transportation as a key component in the fight against the burden of NCDs and climate change, two major health challenges for the LAC region in the 21st century.

#### Disclaimer.

Authors hold sole responsibility for the views expressed in the manuscript, which may not necessarily reflect the opinion or policy of the *RPSP/PAJPH* or the Pan American Health Organization (PAHO).

#### Funding.

LFMR receives doctoral scholarship from the São Paulo Research Foundation (2014/25614-4). THS acknowledges funding from the São Paulo Research Foundation (Fapesp, 2012/08565-4 and 2013/25624-7) and the National Council for Scientific and Technological Development (CNPq, 200358/2014-6 and 402648/2015-3).
